# Analysis of the response time to involved-field radiotherapy in primary gastrointestinal low-grade B-cell lymphoma

**DOI:** 10.1186/s13014-020-01649-6

**Published:** 2020-08-31

**Authors:** Kyu Hye Choi, Han Hee Lee, Seung-Eun Jung, Kyung-Sin Park, Joo-Hyun O, Young-Woo Jeon, Byung-Ock Choi, Seok-Goo Cho

**Affiliations:** 1 Department of Radiation Oncology, Catholic University Lymphoma Group, Seoul St. Mary’s Hospital, College of Medicine, The Catholic University of Korea, Seoul, Republic of Korea; 2 Department of Gastroenterology, Catholic University Lymphoma Group, Yeouido St. Mary’s Hospital, College of Medicine, The Catholic University of Korea, Seoul, Republic of Korea; 3 Department of Radiology, Catholic University Lymphoma Group, Eunpyeong St. Mary’s Hospital, College of Medicine, The Catholic University of Korea, Seoul, Republic of Korea; 4 Department of Pathology, Catholic University Lymphoma Group, Seoul St. Mary’s Hospital, College of Medicine, The Catholic University of Korea, Seoul, Republic of Korea; 5 Department of Nuclear Medicine, Catholic University Lymphoma Group, Seoul St. Mary’s Hospital, College of Medicine, The Catholic University of Korea, Seoul, Republic of Korea; 6 Department of Hematology, Catholic University Lymphoma Group, Yeouido St. Mary’s Hospital, College of Medicine, The Catholic University of Korea, Seoul, Republic of Korea; 7 Department of Hematology, Catholic University Lymphoma Group, Seoul St. Mary’s Hospital, College of Medicine, The Catholic University of Korea, Seoul, Republic of Korea

## Abstract

**Background:**

Early-stage primary gastrointestinal (GI) low-grade B-cell lymphoma shows good therapeutic response to primary radiotherapy. However, there is no clear guideline for the evaluation of response to radiation therapy currently. The aim of this study was to analyze the relationship between the best response time and the clinical course after radiotherapy.

**Methods:**

Patients who underwent radiotherapy for treatment of primary GI low-grade B-cell lymphoma from September 2007 to December 2018 at Seoul St. Mary’s Hospital were included. Early responders were defined by best response within 6 months after radiotherapy, and delayed responders after 6 months. Clinical and pathological factors associated with delayed response and survival analyses were performed to investigate the recurrence and survival during follow-up.

**Results:**

A total of 43 patients were evaluated and the number of gastric mucosa-associated lymphoid tissue and duodenal follicular lymphoma was 36 and 7, respectively. All of 43 patients showed complete remission to radiotherapy and the best response time after radiotherapy was a median of 3 months. There were 8 delayed responders with a median duration of 8.9 months. Early and delayed responders were characterized by a significant difference in depth of invasion beyond the mucosal layer.

**Conclusions:**

Delayed responders did not show differences in oncological outcomes compared with early responders. They were allowed to watch and wait for an additional 6 to 12 months without further treatment.

## Background

Gastrointestinal (GI) lymphoma is the most common extranodal lymphomas and primary GI lymphoma is usually diagnosed in the stomach, duodenum, esophagus and rectum [1, 2]. More than 90% of all GI lymphomas are of B-cell lineage and treatment modalities differ according to the pathological subtype [3]. Systemic involvement of B-cell lymphomas in early stage with low-grade pathology such as mucosa-associated lymphoid tissue (MALT) or follicular lymphoma is rare and it can be cured by local radiotherapy or observed without treatment.

Factors known to cause primary GI lymphoma include bacterial infections such as *H.pylori* and *C.jejuni*, preexisting intestinal diseases, and immunosuppression [4]. In the case of gastric lymphoma infected with *H.pylori*, *H.pylori* eradication (HPE) consisted of proton pump inhibitors, amoxicillin, and clarithromycin is the primary treatment resulting in an efficacy of over 80% [5, 6]. Involved-field radiotherapy (IFRT) is indicated for patients with *H.pylori*-positive lymphoma refractory to HPE as the second-line treatment or *H.pylori*-negative lymphoma as first-line [7, 8]. Gastric lymphoma tends to be a multifocal disease, so the entire stomach should be irradiated according to the International Lymphoma Radiation Oncology Group (ILROG) guidelines [9].

Primary GI low-grade follicular lymphoma is predominantly found in the second portion of the duodenum, with polypoid lesions appearing sporadically in the affected organs. Early-stage GI follicular lymphoma has been monitored without any treatments in the past; however, radiotherapy has been administered to patients with GI follicular lymphoma because of the relatively better overall survival rate [10]. The dose of ≤30 Gy showed an effective local control rate of ≥90%. And ILROG also recommend ≥24 Gy as radiation dose of early stage GI low-grade follicular lymphoma, and the entire duodenum should be included as radiotherapy field [9, 11]. Recently, options for radiation doses of less than 4 Gy (2 × 2 Gy) in recurrent follicular lymphoma have also been proposed and have shown an acceptable response rate of 92% [12]. However, as a rare disease, there is no standardized guideline for the duration and frequency of follow-up in patients receiving radiotherapy.

Clinical and endoscopic factors associated with delayed response after radiotherapy or the timing of follow-up in primary GI low-grade B-cell lymphoma (PG-LGBCL) have not been studied and the protocol of follow-up varies from institution to institution. This study was conducted to analyze the duration of the response and delayed responders to radiotherapy in PG-LGBCL at a single institution. The purpose of this study was to determine the interval of follow-up needed and avoid unnecessary additional treatment.

## Materials and methods

Patients with PG-LGBCL treated with curative radiotherapy at Seoul St. Mary’s Hospital from September 2007 to December 2018 were analyzed retrospectively. Patients underwent blood tests, endoscopy, endoscopic biopsy, abdominal CT, and fluorine-18 fluorodeoxyglucose (^18^F-FDG) positron emission tomography (PET) at initial diagnosis. Endoscopic ultrasound (EUS) to confirm the depth of the lesion was performed in stomach lymphoma patients. This study was approved by the ethical review board (Seoul St. Mary’s Hospital, College of Medicine, The Catholic University of Korea, reference number: KC19RESI0121).

**Fig. 1:**
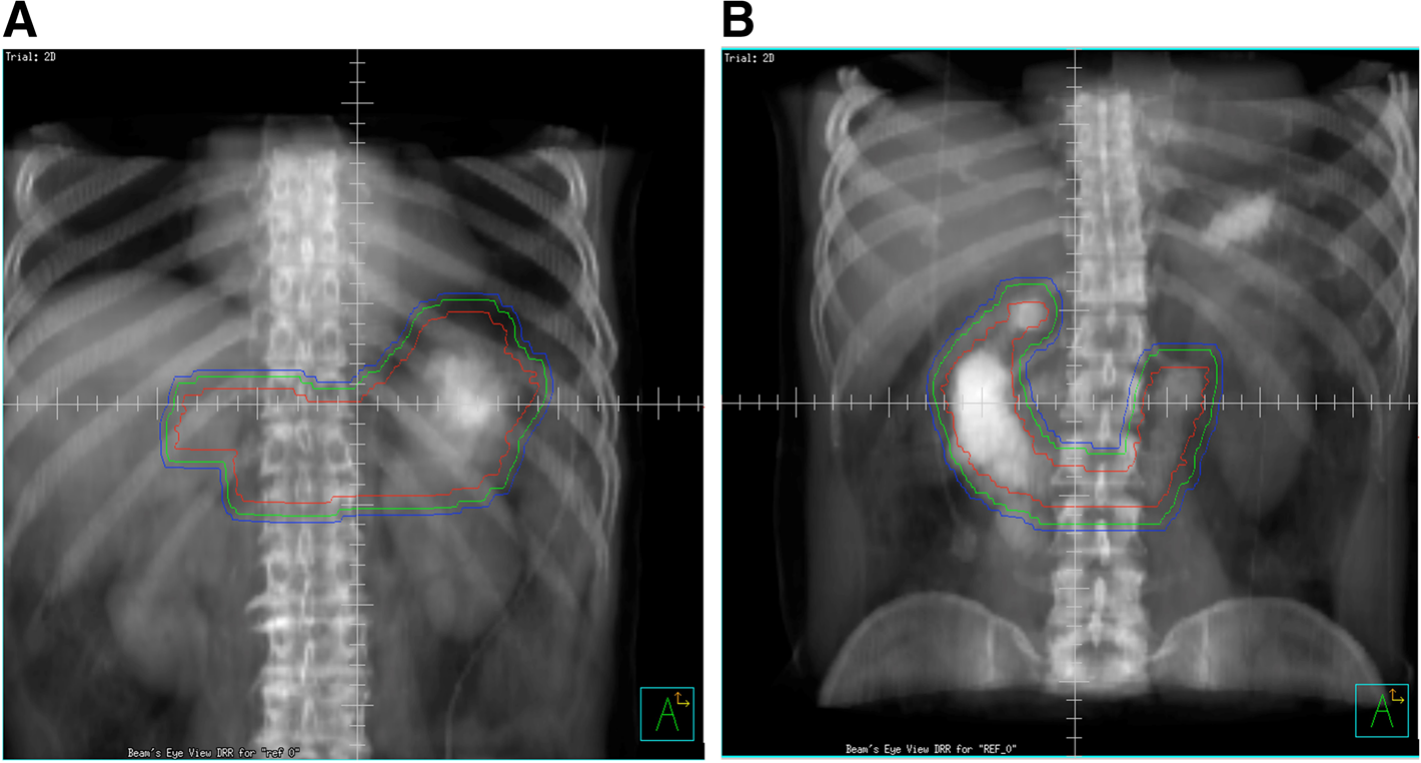
Images presenting examples of CTV (red-colored line), ITV (green-colored) and PTV (blue-colored) in this study (**a**, stomach; **b**, duodenum). CTV, clinical target volume; ITV, internal target volume; PTV, planning target volume

Patients completed treatment courses of IFRT, and patients involved ≥2 organs were excluded. The clinical target volume (CTV) included the entire involved organ and the regional lymph nodes if involved. The internal target volume (ITV) was set using the motion information obtained from the 4-dimensional CT for assessment of breathings and abdominal motions. Planning target volume (PTV) was defined by expanding 5 mm from ITV considering the patient’s set-up error. Radiotherapy was prescribed as 30.6 Gy over 17 fractions on stomach or 24 Gy over 12 fractions on duodenum. Images presenting an example of CTV, ITV and PTV are illustrated in Fig. 1. The dose constraints to organ at risk used for radiotherapy planning were as follows: the maximum dose (Dmax) to spinal cord < 40 Gy, Dmax of the duodenum < 50 Gy, V50 of the small intestine < 5%, V20 of both kidneys < 20%, and V30 of the liver < 30% (Vx, the percentage of organ receiving x Gy).

After treatment completion, patients underwent CT, endoscopy and biopsy every 3 months for the first year, every 6 months for the next 5 years, and then annually. The complete remission (CR) was pathologic and radiologic remission in endoscopic biopsy and imaging. The first CR of the follow-up was defined as the best response. The duration of best response was the time period between the completion of radiotherapy and the best response. The best response within 6 months after radiotherapy was defined as early response and that after > 6 months was defined as delayed response. The National Comprehensive Cancer Network (NCCN) guideline recommends evaluation of the lesion at 3 to 6 months in patients diagnosed with PG-LGBCL. The hypothesis of this study was that clinical outcomes of delayed responders after > 6 months may differ from those of early responders.

During the follow-up period, we analyzed the recurrence and survival rates of patients using Kaplan-Meier survival analysis and evaluated the clinical and pathological factors of delayed responders using multivariate logistic regression analysis. *P*-values less than 0.05 were considered statistically significant. Statistical analysis was performed using SPSS software version 24 (SPSS Inc., Chicago, IL, U.S.A.). Furthermore, the medical records were reviewed in accordance with the National Cancer Institute’s Common Terminology Criteria for Adverse Events (NCI CTCAE) version 5.0 to investigate the acute and chronic side effects of radiotherapy.

## Results

A total of 43 patients were included in this study and the median follow-up period was 27.9 months (range, 8.8–117 months). The clinical characteristics of 43 patients are summarized in Table 1. They comprised 36 gastric MALT lymphomas and 7 duodenal follicular lymphomas, and clinical stage of all patients was Lugano I or II_1_. Fourteen of gastric lymphoma patients were refractory to the eradication therapy in *H.pylori* infection and received radiotherapy as the second-line treatment. The other 22 gastric lymphomas patients with negative for *H.pylori* infection were treated with radiotherapy as primary treatment. Five patients were tested for BIRC3/MALT1 translocation before treatment, one of which yielded positive results. Seven patients in duodenal lesions were follicular lymphomas and all received radiotherapy as the primary treatment. The treatment technique included 3-dimensional conformal radiotherapy and intensity-modulated radiotherapy in 38 and 5 patients, respectively.

**Table 1:** The clinical and tumor characteristics of 43 patients

Characteristics		N or median
Age (years old) (median, range)		56 (36–84)
Sex (N, %)	Male	20 (46.5)
	Female	23 (53.5)
Initial stage (N, %)	Lugano I	38 (88.4)
	Lugano II_1_	5 (11.6)
Initial IPI (N, %)	0	28 (65.1)
	1	15 (34.9)
Initial largest size (cm) (median, range)		2.4 (0.3–6)
Primary site (N, %)	Stomach	36 (83.7)
	Duodenum	7 (16.3)
*H.pylori* infection (N, %)	Negative	29 (67.4)
	Positive	14 (32.6)
Genetic alteration (N, %)	Yes	1 (2.3)
	No	4 (9.3)
	N/A	38 (88.4)
Multiplicity	Single	11 (25.6)
	Multiple	32 (74.4)
Initial nodal involvement (N, %)	Yes	5 (11.6)
	No	38 (88.4)

*IPI* International prognostic index; *N/A* not available

All of 43 patients achieved pathologic CR after radiotherapy with a median duration of 3 months (range, 2.1–14.6 months). Radiologic or metabolic CR in CT or PET-CT was also demonstrated in regional node metastasis of 5 patients. Thirty-five patients showed the best responses in the endoscopy and biopsy at the first follow-up within 6 months. Eight patients were delayed responders, and their median duration of best response was 8.9 months (range, 6.1–14.6 months). Of 8 delayed responders, five patients had CR at the second follow-up (6.1–9 months) after completion of treatment and 3 patients at the third follow-up (9.7–14.6 months). Thirty-five patients who had CR within 6 months and 8 patients after > 6 months were classified into early and delayed responder groups, respectively.

**Fig. 2:**
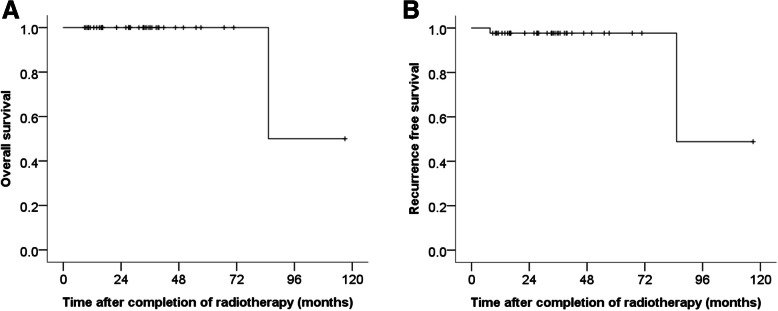
Kaplan-Meier curves for OS (**a**) and RFS (**b**) of the 43 patients. OS, overall survival; RFS, recurrence-free survival

One patient received salvage chemotherapy during follow-up period because of other organ involvement out of radiation field 7.7 months after completion of radiotherapy. She was a patient with early response of pathologic CR to radiotherapy at the first-line treatment. Another patient died at 7 years because of lymphoma-unrelated cause. The 2-year overall survival (OS) and recurrence-free survival (RFS) rates were 100 and 97.1%, respectively. The 5-year OS and RFS rates were 100 and 97.1%, respectively. Figure 2 shows Kaplan-Meier curves for OS and RFS of the 43 patients. The OS and RFS between two responder groups were not significantly different (*P* = 0.633). Grade 1 and 2 GI toxicity according to NCI CTCAE version 5.0 was observed in 12 patients (27.9%) and 6 patients (14.0%), respectively. Their main complaints were nausea or epigastric discomfort and improved after conservative treatment. No adverse events of grade 3 or higher were noted, and there were no stomach or duodenal ulcers or perforations.

Multivariate logistic regression analysis to evaluate the factors associated with significant differences between 35 early responders and 8 delayed responders was conducted (Table 2). There was no significant difference in the pathologic type or tumor location between the early and delayed responders. The lactate dehydrogenase (LDH), Lugano stage, IPI score, and SUV uptake in PET-CT before treatment did not differ significantly between the two groups. The endoscopic and EUS factors related to response to radiotherapy were analyzed (Table 3). There was no significant difference in location, size, multiplicity, or endoscopic pattern of the tumor. EUS was performed in 30 out of 36 gastric lymphoma patients, and there was statistically significant difference of deep invasion beyond the mucosal layer in EUS between two responder groups (*P* = 0.027). The odds ratio was 0.400 and 95% confidence interval was 0.155–1.031.

**Table 2:** Multivariate logistic regression analysis to assess clinical factors affecting the duration of best response

Characteristics	Early responder (*n* = 35)	Delayed responder (*n* = 8)	*P*-value^a^
Age			0.255
≤ 60 years old (*n* = 29)	25 (71.4)	4 (50.0)	
> 60 years old (*n* = 14)	10 (28.6)	4 (50.0)	
Sex			0.167
Male (*n* = 20)	18 (51.4)	2 (25.0)	
Female (*n* = 23)	17 (48.6)	6 (75.0)	
Initial LDH ≥500 U/L			0.302
< 500 U/L (*n* = 41)	34 (97.1)	7 (87.5)	
≥ 500 U/L (n = 2)	1 (2.9)	1 (12.5)	
Initial Lugano staging			0.230
Stage I (*n* = 38)	32 (91.4)	6 (75.0)	
Stage II_1_ (*n* = 5)	3 (8.6)	2 (25.0)	
Initial IPI score			0.076
0 (*n* = 28)	25 (71.4)	3 (37.5)	
1 (*n* = 15)	10 (28.6)	5 (62.5)	
Initial ^18^F-FDG uptake SUVmax ≥3			0.328
< 3 (*n* = 24)	20 (57.1)	4 (50.0)	
≥ 3 (*n* = 13)	9 (25.7)	4 (50.0)	
N/A (*n* = 6)	6 (17.1)	0	
Initial nodal involvement			0.230
No (n = 38)	32 (91.4)	6 (75.0)	
Yes (n = 5)	3 (8.6)	2 (25.0)	
Primary tumor site			0.479
Stomach (*n* = 36)	30 (85.7)	6 (75.0)	
Duodenum (*n* = 7)	5 (14.3)	2 (25.0)	
*H.pylori* infection			0.255
Negative (n = 29)	25 (71.4)	4 (50.0)	
Positive (n = 14)	10 (28.6)	4 (50.0)	

*LDH* lactate dehydrogenase; *IPI* International prognostic index; ^*18*^*F-FDG* ^18^F-fluorodeoxyglucose; *SUVmax* maximum standardized uptake value; *N/A* not available

^a^ Multivariate logistic regression analysis

**Table 3:** Multivariate logistic regression analysis to assess endoscopic factors affecting the duration of best response

Endoscopic findings	Early responder (n = 35)	Delayed responder (n = 8)	*P*-value^a^
Tumor location on endoscopy			0.728
Fundus, cardina (n = 6)	4 (11.4)	2 (25.0)	
Body (*n* = 18)	16 (45.7)	2 (25.0)	
Antrum, pylorus (n = 4)	3 (8.6)	1 (12.5)	
Duodenal 2nd portion (n = 5)	4 (11.4)	1 (12.5)	
Duodenal 3rd portion (n = 2)	1 (2.9)	1 (12.5)	
Multi-section in single organ (n = 8)	7 (20.0)	1 (12.5)	
Initial size on endoscopy ≥2 cm			0.370
< 2 cm (n = 8)	7 (20.0)	1 (12.5)	
≥ 2 cm (n = 14)	10 (28.6)	4 (50.0)	
N/A (*n* = 21)	18 (51.4)	3 (37.5)	
Multiplicity			0.967
Single (*n* = 11)	9 (25.7)	2 (25.0)	
Multiple (*n* = 32)	26 (74.3)	6 (75.0)	
Endoscopic pattern			0.594
Erythema (n = 4)	4 (11.4)	0	
Erosion (n = 1)	1 (2.9)	0	
Nodularity (n = 20)	15 (42.9)	5 (62.5)	
Ulceration (*n* = 10)	8 (22.9)	2 (25.0)	
Atrophy (n = 8)	7 (20.0)	1 (12.5)	
Depth on EUS			0.083
Mucosa to MM (*n* = 9)	9 (25.7)	0	
SM (n = 15)	11 (31.4)	4 (50.0)	
PM (n = 6)	4 (11.4)	2 (25.0)	
N/A (n = 13)	11 (31.4)	2 (25.0)	
Depth within the mucosal layer^b^			0.027
Within the mucosal layer (n = 9)	9 (37.5)	0	
Beyond the mucosal layer (n = 21)	15 (62.5)	6 (100)	

*EUS* endoscopic ultrasound; *MM* muscularis mucosa; *SM* submucosa; *PM* proper muscle; *N/A* not available

^a^ Multivariate logistic regression analysis

^b^ 13 patients were not available

## Discussion

PG-LGBCL in early stage can be cured with local radiotherapy, and this study resulted that pathologic CR after IFRT was achieved in all patients, suggesting radiation dose of 25–30 Gy showed adequate tumor control. Previous studies also reported a high response rate of 90% to radiotherapy in cases diagnosed with PL-LGBCL, with RFS rate of 90–100% and an OS rate of 75–100% [13–17]. A recent retrospective study analyzed the efficacy of the radiotherapy field reduction in low-grade follicular and MALT lymphoma, and reported that there was no difference in local control and survival between the existing IFRT and involved-site radiotherapy [18]. Further research is underway to reduce radiation fields and doses in low-grade B-cell lymphoma [19, 20]. However, the clinical factors that affect the response time and oncologic outcome of delayed response to radiation therapy have not yet been evaluated in previous studies.

The current study demonstrated that local tumor control was good even with delayed response to radiotherapy. Only one patient in early responder group developed out-field recurrence and there was no recurrence in delay responder group. This results suggested that the lesion can be cured without further treatment even if the response to radiation therapy was delayed. In addition, initial evaluation of radiotherapy after 6 months or interval increases could reduce unnecessary invasive tests.

For treatment of *H.pylori*-positive gastric lymphoma, eradication therapy using antibiotics is the first-line treatment and highly invasive lymphomas are known to respond later more than 6 months [21, 22]. Previous studies have shown that patients with lymphoma extending beyond the submucosal layer can respond after 1 year of HPE, so endoscopy and biopsy are recommended every 6 months for the first 2 years [23–25]. The heterogeneity of *H.pylori* strain may result in different responses or different tumor location might affect different responses to HPE [21, 26, 27]. Therefore, secondary treatment after eradication therapy is recommended if the gross lesion persists for 6 months and microscopic lymphomatous infiltration occurs up to 2 years [28]. A delayed response to eradication therapy indicates poor prognosis warranting secondary treatment such as radiotherapy.

On the other hand, few studies investigating delayed response to radiotherapy have been conducted. Cheson et al. [29] and the NCCN guidelines recommended an evaluation of endoscopy and biopsy at 3 to 6 months after radiotherapy. However, 8 of the 43 patients manifested the delayed response after > 6 months, which may require further follow-up to evaluate the final response to radiotherapy.

Interestingly, clinical and endoscopic factors that affect delayed response after radiation therapy were analyzed in this study. In multivariate logistic regression analysis, the depth of invasion beyond mucosal layer showed a significant difference between two responder groups. Other characteristics such as age, stage, LDH, and IPI score did not show statistical difference between two groups. Previous studies reported differences in eradication therapy depending on sex or tumor location; however, no significant difference in response time of radiotherapy was demonstrated [26, 27]. Although there were delayed response in deeply invasive lymphoma, the patients were observed without salvage treatment and all achieved CRs finally.

The endoscopic findings such as ulcerative pattern indicated the high grade lymphoma in previous study [30]. In the endoscopic findings of current study, there was no significant difference in endoscopic patterns between two responder groups. However, the first endoscopic finding of the 7 patients in 8 delayed responders was characterized by diffuse or deep nodularity and ulceration rather than erosion or erythema at the initial examination. The number of patients was small so there was no difference between endoscopic findings and delayed response.

The aim of the current study was not only to evaluate the response rate but also to analyze the factors associated with the delayed response. The limitation of this study are its retrospective nature with small number of patients, as well as short follow up with median of 27.9 months, and therefore it is difficult to compare the oncological outcome in this short period. A prospective large-scale study is needed to analyze the factors that may cause delayed response and to establish a protocol for evaluation of response to radiotherapy of PG-LGBCL. To evaluate the oncologic outcome and late toxicity results in radiotherapy for PG-LGBCL, longer follow-up will be necessary.

Second, the different prescription doses of duodenal follicular lymphoma and gastric MALT lymphoma show limitations in the dose inhomogeneity of retrospective studies. In the UK, a 3-phase multicenter prospective study was conducted comparing the local control rates of 40 Gy and 24 Gy in indolent lymphoma, and the local control rates were 93% and 92%, respectively, which did not differ between the two groups [19]. In addition, Lee et al. reported 95% complete remission of the duodenal follicular lymphoma after 24 Gy irradiation [20]. Therefore, 24 Gy for duodenal follicular lymphoma and 30.6 Gy for gastric MALT lymphoma were prescribed in our instution, and there was no difference in local control between 24 Gy and 30.6 Gy in this study.

In conclusion, PG-LGBCL showed an adequate response rate, RFS, and OS with limited toxicity with radiotherapy. And there were no significant difference of recurrence and local control between response time to radiotherapy. Furthermore, deep invasion beyond mucosal layer affected delayed response to radiotherapy. This study suggested more studies of factors and prognosis affecting the delayed response. Additional clinical studies are needed, but PG-LGBCL with response to radiation therapy may be followed up without further treatment.

## Data Availability

The dataset used and analyzed during the current study are available from the corresponding author on reasonable request.

## Abbreviations

GI: Gastrointestinal
MALT: Mucosa-associated lymphoid tissue
HPE: *H.pylori* eradication
IFRT: Involved-field radiotherapy
ILROG: International Lymphoma Radiation Oncology Group
PG-LGBCL: Primary GI low-grade B-cell lymphoma
^18^F-FDG: Fluorine-18 fluorodeoxyglucose
PET: Positron emission tomography
EUS: Endoscopic ultrasound
CTV: Clinical target volume
ITV: Internal target volume
PTV: Planning target volume
Dmax: Maximum dose
Vx: the percentage of organ receiving x Gy
CR: Complete remission
NCCN: National Comprehensive Cancer Network
NCI CTCAE: National Cancer Institute’s Common Terminology Criteria for Adverse Events
OS: Overall survival
RFS: Recurrence-free survival
LDH: Lactate dehydrogenase
